# Effect of the sodium–glucose cotransporter 2 inhibitor luseogliflozin on pancreatic beta cell mass in db/db mice of different ages

**DOI:** 10.1038/s41598-018-25126-z

**Published:** 2018-05-01

**Authors:** Kiyohiko Takahashi, Akinobu Nakamura, Hideaki Miyoshi, Hiroshi Nomoto, Naoyuki Kitao, Kazuno Omori, Kohei Yamamoto, Kyu Yong Cho, Yasuo Terauchi, Tatsuya Atsumi

**Affiliations:** 1 Department of Rheumatology, Endocrinology and Nephrology, Faculty of Medicine and Graduate School of Medicine, Hokkaido University, Sapporo, Japan; 2 Department of Endocrinology and Metabolism, Graduate School of Medicine, Yokohama City University, Yokohama, Japan

## Abstract

To examine the effects of luseogliflozin, a sodium–glucose cotransporter 2 inhibitor, on pancreatic beta cell mass in db/db mice of different ages. db/db mice aged 6, 10, 14 and 24 weeks old were fed either standard chow (control group) or standard chow containing 0.01% luseogliflozin (luseo group). After 4 weeks, immunohistochemistry and gene expression tests were conducted. In 6-week-old db/db mice, immunohistochemistry revealed a significant increase in beta cell mass in the luseo group compared with the control group after 4 weeks of treatment. Gene expression profiling of isolated islets showed upregulation *Mafa*, *Pdx1*, *Ki67* and *Ccnd2* in the luseo group. Beta cell mass decreased with age in db/db mice in the control group. Beta cell mass in the luseo group significantly increased compared with the control group regardless of age, although beta cell mass in the 28-week-old luseo group (4 weeks of treatment in 24-week-old db/db mice) was significantly lower than in the 10-week-old luseo group (4 weeks of treatment in 6-week-old db/db mice). Luseogliflozin preserved beta cell mass in db/db mice. The protective effect was more evident in the earlier phase of diabetes.

## Introduction

Type 2 diabetes mellitus (T2D) is characterized by insulin resistance in tissues including the liver, skeletal muscle and adipose tissue, and by impaired pancreatic beta cell function^1^. To maintain normal blood glucose with insulin resistance, beta cell mass and/or insulin secretion increase as a compensatory mechanism. Beta cell mass in T2D is insufficient to compensate for insulin demands^2^. Human studies suggest that beta cell mass in patients with T2D, regardless of body mass, decreases compared with healthy subjects^3,4^, and obese patients with impaired fasting glucose also show decreased beta cell mass^3^. The United Kingdom Prospective Diabetic Study has suggested that deterioration of pancreatic beta cell function becomes apparent several years before a diagnosis of T2D and is part of the natural history of T2D progression^5^.

The db/db mice carry a deleterious point mutation in the leptin receptor gene. This animal is used as a model of T2D, showing both obesity and increased insulin resistance. Beta cell mass in db/db mice declines with advancing age^6,7^. Early protection of pancreatic beta cells is crucial for preventing both beta cell loss and dysfunction.

Sodium–glucose cotransporter 2 (SGLT2) inhibitors improve glucose tolerance by suppressing renal glucose reabsorption without direct pharmacological action on pancreatic beta cells^8–11^. The absence of SGLT2 in db/db mice prevented a reduction in beta cell mass and preserved glucose-stimulated insulin secretion^12^. Chronic treatment with an SGLT2 inhibitor for 4 weeks has been reported to increase beta cell mass in 10-week-old db/db mice^13^. Nevertheless, the effects of SGLT2 inhibitors on beta cell mass in db/db mice at different diabetic stages are unknown.

The objective of this study was to characterize the *in vivo* effects of luseogliflozin, an SGLT2 inhibitor, on pancreatic beta cell mass and function in db/db mice. Moreover, to investigate the protective effects of luseogliflozin on pancreatic beta cells not only in the early phase of diabetes, but also in the late phase of diabetes, these effects in db/db mice of different ages were also compared.

## Results

### Effects of luseogliflozin on metabolic changes and glucose tolerance in 6-week-old db/db mice

**Figure 1:**
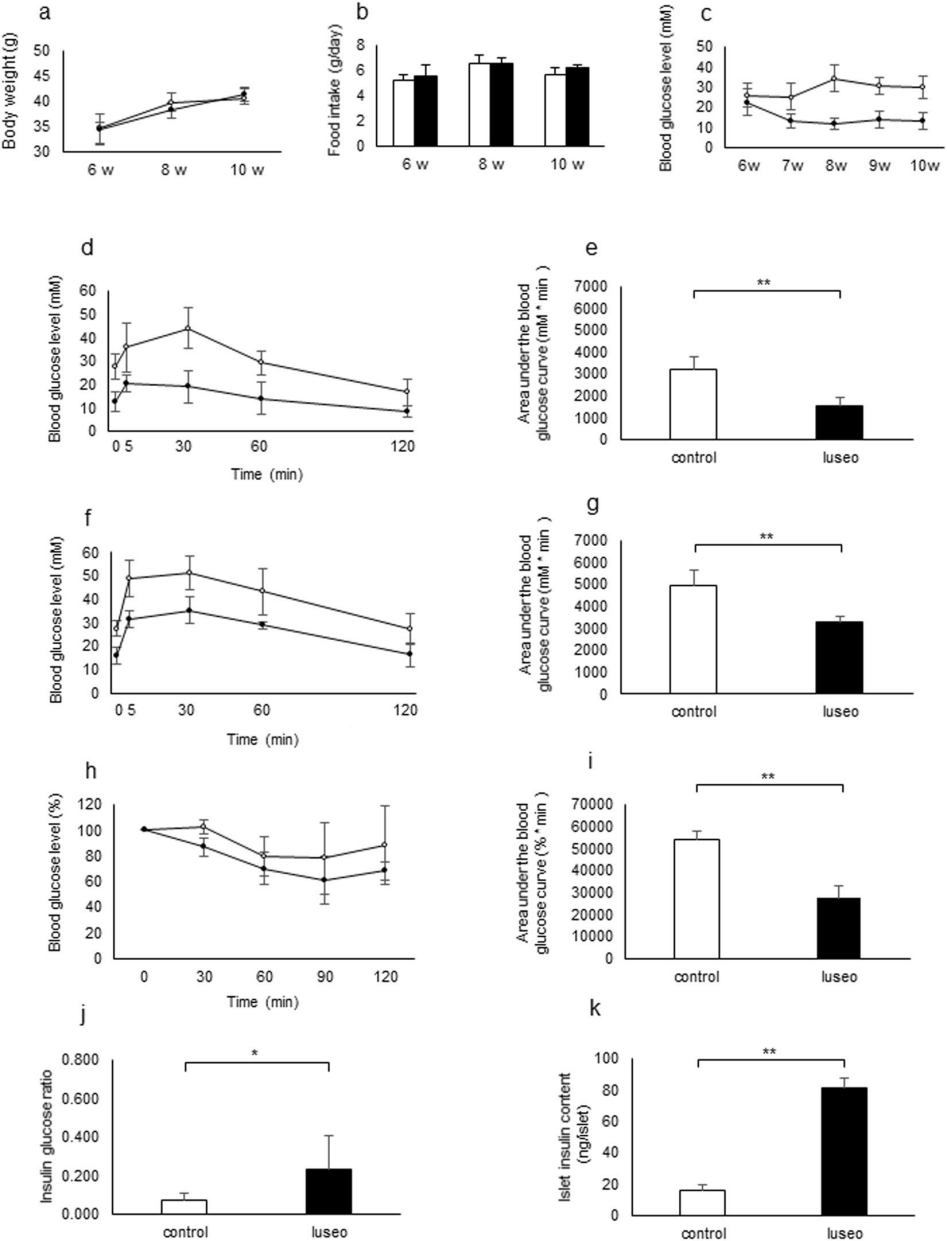
Effects of luseogliflozin on metabolic changes and glucose tolerance in 6-week-old db/db mice. (**a–c**) Changes in (**a**) body weight, (**b**) food intake, and (**c**) blood glucose levels in 6-week-old db/db mice fed standard chow (control group; white circles) or standard chow containing luseogliflozin 0.01% (luseo group; black circles) for 4 weeks (*n* = 5–12). (**d**) Blood glucose levels during the oral glucose tolerance test (OGTT) in the control group (white circles) and the luseo group (black circles) after a 4-h fast in 10-week-old db/db mice (*n* = 8). (**e**) Area under the curve for glucose excursion during the OGTT in the control group (white bar) and the luseo group (black bar) after a 4-h fast in 10-week-old db/db mice (*n* = 8). (**f**) Blood glucose levels during the OGTT in the control group (white circles) and the luseo group (black circles) after a 16-h fast in 10-week-old db/db mice (*n* = 8). (**g**) Area under the curve for glucose excursion during the OGTT in the control group (white bar) and luseo group (black bar) after a 16-h fast in 10-week-old db/db mice (*n* = 8). (**h**) Blood glucose levels during the intraperitoneal insulin tolerance test in the control group (white circles) and the luseo group (black circles) in 10-week-old db/db mice (*n* = 4). (**i**) Area under the curve for blood glucose levels during the intraperitoneal insulin tolerance test in the control group (white bar) and the luseo group (black bar) in 10-week-old db/db mice (*n* = 4). (**j**) Fasting plasma insulin/glucose ratio in the control group (white bar) and the luseo group (black bar) in 10-week-old db/db mice (*n* = 8). (**k**) Islet insulin content in the control group (white bar) and the luseo group (black bar) in 10-week-old db/db mice (*n* = 6). Values are mean ± SD. *p < 0.05; **p < 0.01.

To determine the effects of luseogliflozin on body weight and glucose levels, we divided 6-week-old db/db male mice into two groups: db/db mice fed standard chow (control group); and db/db mice fed standard chow containing 0.01% luseogliflozin (luseo group) for 4 weeks. Although there were differences neither in either body weight nor in food intake between the two groups after 4 weeks’ treatment (Fig. 1a,b), blood glucose levels significantly decreased in the luseo group compared with the control group (Fig. 1c). Both groups underwent an OGTT after a 4-h fast and the AUC_0–120 min_ for blood glucose significantly decreased in the luseo group compared with the control group (Fig. 1d,e). To investigate whether the 4-week’s treatment with luseogliflozin *per se* might have contributed to the improvement in glucose tolerance, we performed an OGTT at 16 h after discontinuation of luseogliflozin administration. Similarly, the AUC_0–120 min_ in the luseo group showed significantly improved glucose tolerance compared with the control group (Fig. 1f,g). To examine the effects of luseogliflozin on insulin resistance in db/db mice, we performed an intraperitoneal insulin tolerance test and found that insulin resistance improved in the luseo group compared with the control group (Fig. 1h,i). To assess the effects of luseogliflozin on beta cell function, we measured fasting plasma insulin and insulin content in pancreatic islets. Both the ratio of insulin/glucose and insulin content of pancreatic islets in the luseo group were significantly higher than those in the control group (Fig. 1j,k). These results indicated that luseogliflozin administration for 4 weeks improved insulin resistance and pancreatic beta cell function, resulting in the amelioration of glucose tolerance in 6-week-old db/db mice.

### Effects of luseogliflozin on beta cell morphology in 6-week-old db/db mice

**Figure 2:**
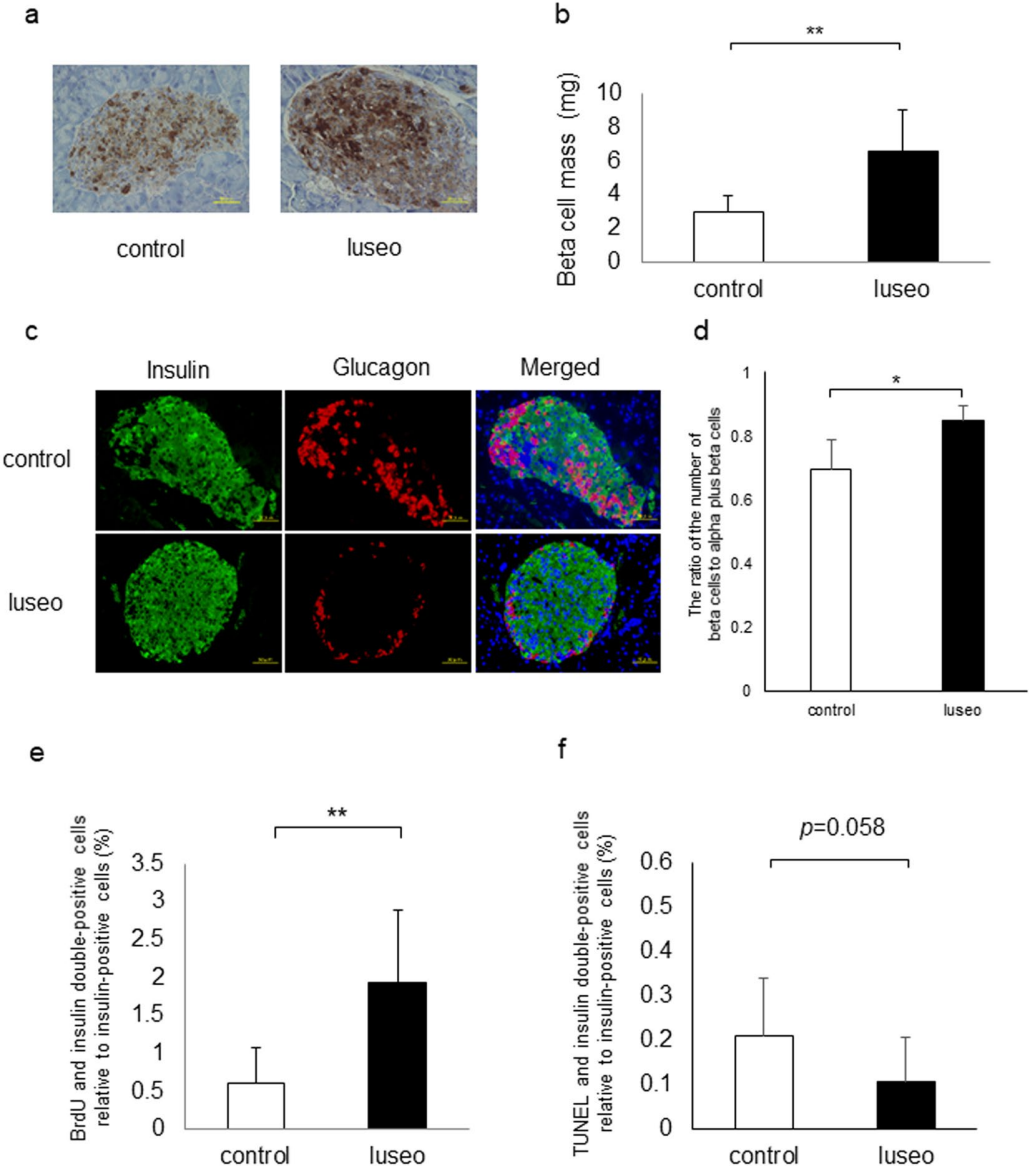
Impact of luseogliflozin on beta cell morphology in 6-week-old db/db mice. (**a)** Histological analysis of pancreatic islets of db/db mice with or without luseogliflozin for 4 weeks. Beta cells are stained brown. (**b**) Quantitation of beta cell mass in the control group (white bar) and the luseo group (black bar) (*n* = 8: eight mice were used in each group). (**c**) Representative insulin (green) and glucagon (red) staining in pancreas sections from the control group and the luseo group. (**d**) The ratio of the number of beta cells to the sum of alpha plus beta cells in pancreatic islets in the control group (white bar) and luseo group (black bar) (*n* = 5: five mice were used in each group). (**e**) Proliferation rate of beta cells assessed using BrdU incorporation in the control group (white bar) and the luseo group (black bar) (*n* = 8: eight mice were used in each group). (**f**) Apoptosis rate of beta cells assessed using the TUNEL assay in the control group (white bar) and the luseo group (black bar) (*n* = 10: ten mice were used in each group). Values are mean ± SD. *p < 0.05; **p < 0.01. Scale bars: 50 μm.

We investigated the effects of luseogliflozin on beta cell mass after 4 weeks treatment. Beta cell mass in the luseo group significantly increased compared with the control group (Fig. 2a,b). We then performed double immunostaining using anti-insulin and anti-glucagon antibodies. In the control group, some alpha cells were observed to have infiltrated the center of islets (Fig. 2c). In the luseo group, beta cells were centrally located and alpha cells were primarily located at the periphery of islets (Fig. 2c). When we calculated the ratio of the number of beta cells to alpha plus beta cells in pancreatic islets, the ratio in the luseo group significantly increased compared with the control group (Fig. 2d). We evaluated beta cell proliferation using immunohistochemical detection of BrdU. The ratio of BrdU-positive beta cells in the luseo group showed a significantly increase compared with the control group (Fig. 2e). The apoptotic activity of beta cells in the luseo group, analyzed using the TUNEL assay, decreased by 50.2% compared with the control group, but not significantly (Fig. 2f).

### Effects of luseogliflozin on changes in gene expression of islets in 6-week-old db/db mice

**Figure 3:**
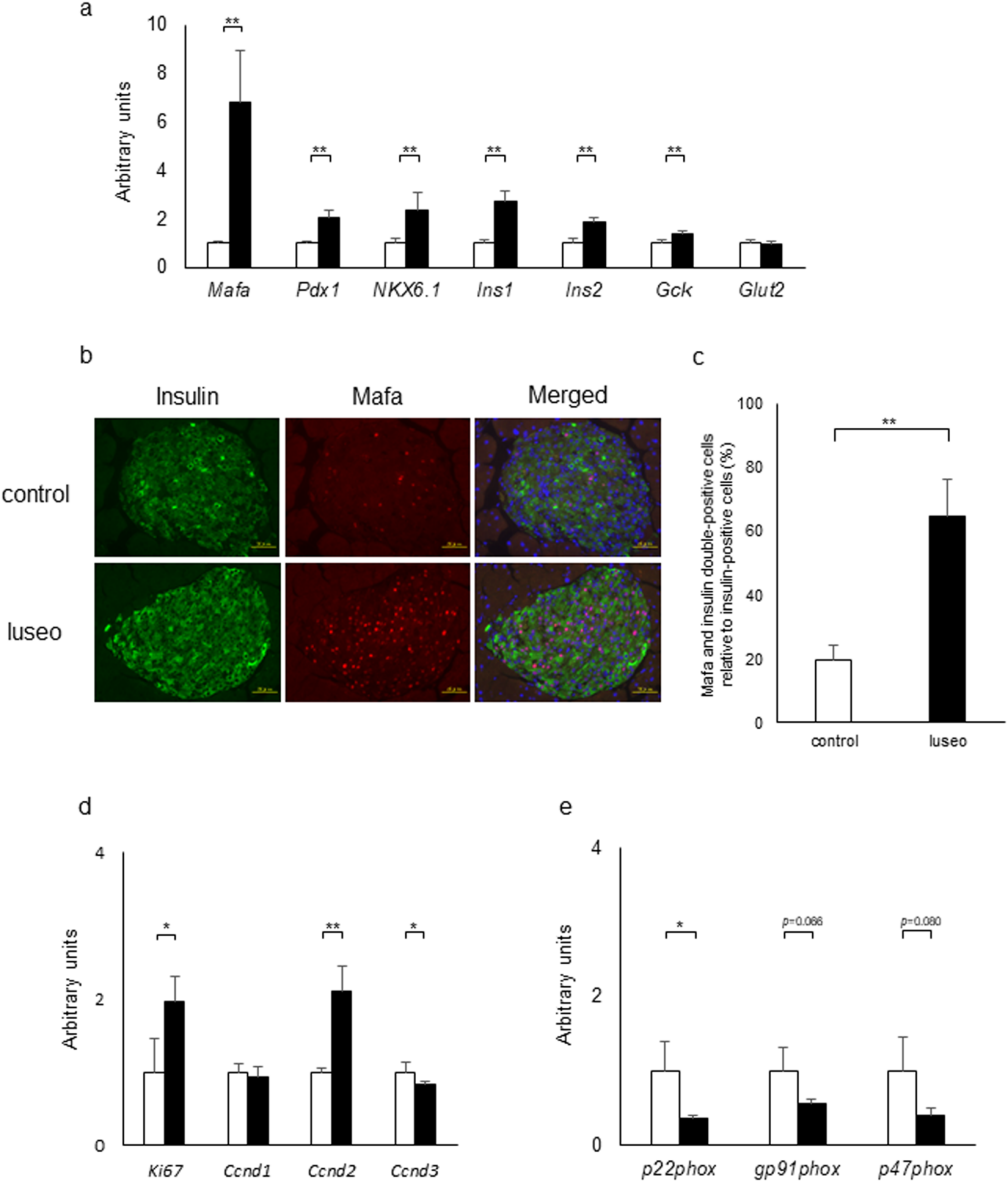
Changes in gene expression levels in islets of 6-week-old db/db mice with or without luseogliflozin for 4 weeks. (**a**) Gene expression levels of *Mafa*, *Pdx1*, *NKX6*.*1*, *Ins1*, *Ins2*, *Gck* and *Glut2* in islets measured using real-time quantitative PCR. Data have been normalized to GAPDH expression. (**b**) Representative insulin (green) and Mafa (red) staining in pancreas sections from the control group and the luseo group. (**c**) The ratio of the number of Mafa-positive beta cells relative to the total number of beta cells in the control group (white bar) and the luseo group (black bar) (*n* = 4: four mice were used in each group). (**d**,**e**) Gene expression levels of *Ki67*, *Ccnd1*, *Ccnd2*, *Ccnd3*, *p22phox*, *gp91phox* and *p47phox* in islets measured using real-time quantitative PCR. Data have been normalized to GAPDH expression. Values are mean ± SD. *p < 0.05; **p < 0.01. Scale bars: 50 μm.

We investigated changes in expression levels of genes associated with beta cell function in islets of db/db mice with or without luseogliflozin. The mRNA expression levels of *Mafa*, *Pdx1*, *NKX6*.*1* and *Gck* (encoding glucokinase) significantly increased in the luseo group compared with the control group (Fig. 3a). Expression levels of *Ins1* and *Ins2*, which are target genes associated with *Mafa* and *Pdx1*, significantly increased in the luseo group compared with the control group (Fig. 3a). Immunohistochemistry revealed that the percentage of Mafa-positive beta cells significantly increased in the luseo group compared with the control group (Fig. 3b,c), being consistent with the results of quantitative real-time PCR. These findings suggested that luseogliflozin improved beta cell function at the transcription level.

We investigated expression levels of genes associated with cell proliferation and the cell cycle. Expression levels of *Ki67* and *Ccnd2* in the luseo group, but not *Ccnd1* or *Ccnd3*, significantly increased compared with the control group (Fig. 3d). Because we had previously found increased expression of NADPH oxidase complex, a marker of oxidative stress, in db/db mice compared with wild-type mice, the expression of genes associated with this complex were evaluated^14^. *P22phox* mRNA levels were significantly lower in the luseo group compared with the control group, and *gp91phox* and *p47phox* mRNA levels tended to be lower in the luseo group compared with the control group (Fig. 3e). Because the accumulation of oxidative stress in beta cells decreases beta cell mass and function^15^, these results suggested that luseogliflozin reduced oxidative stress in the islets of db/db mice, ultimately improving beta cell function and increase beta cell mass.

### Effects of luseogliflozin on metabolic changes and glucose tolerance in db/db mice of different ages

**Figure 4:**
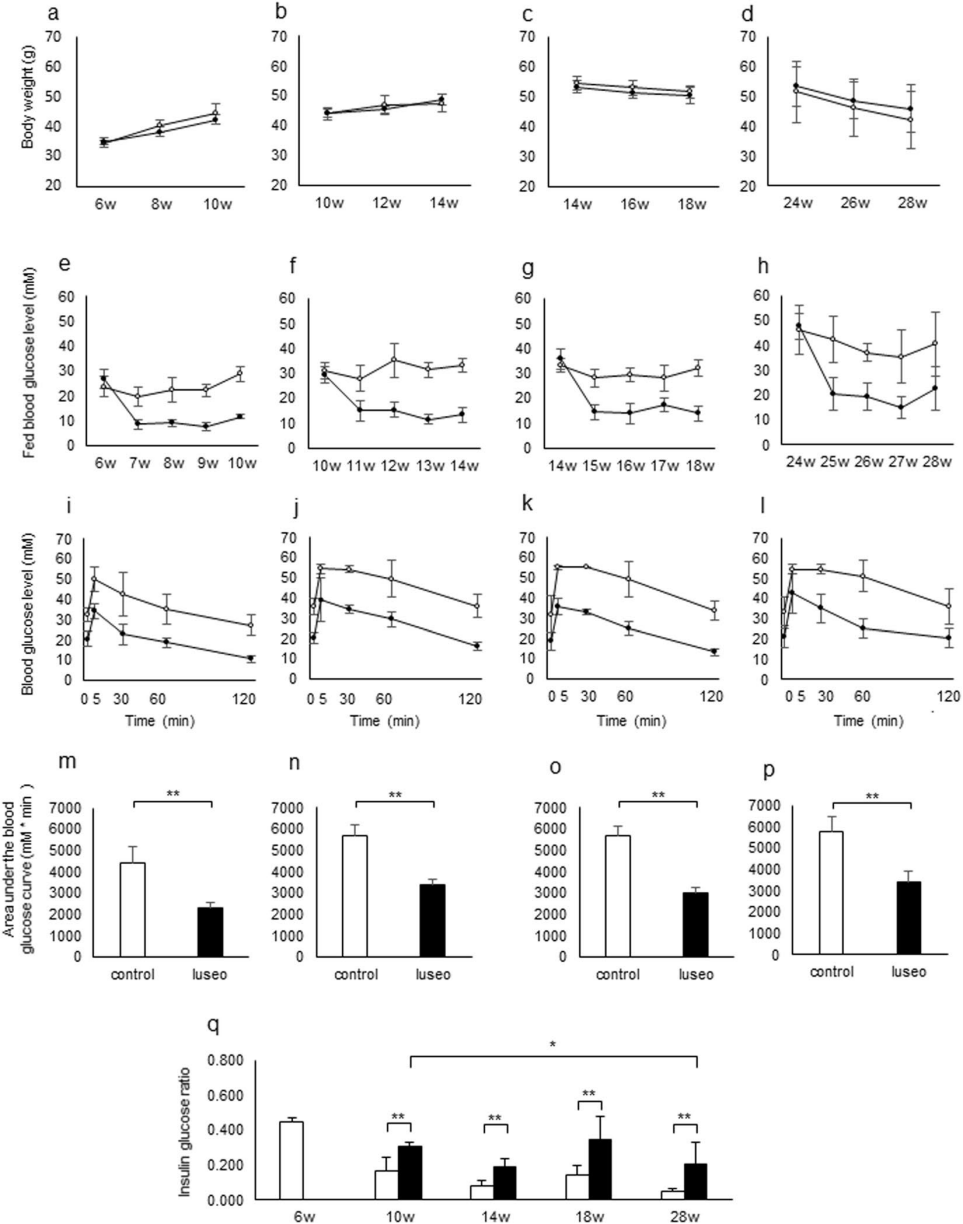
Effect of luseogliflozin on metabolic changes and glucose tolerance in db/db mice of different ages. (**a–h**) Changes in (**a–d**) body weight and (**e–h**) blood glucose levels in 6-, 10-, 14- and 24-week-old db/db mice fed standard chow (control group; white circles) or standard chow containing luseogliflozin 0.01% (luseo group; black circles) for 4 weeks (*n* = 6–8). (**i–l**) Blood glucose levels during the oral glucose tolerance test (OGTT) in the control group (white circles) and the luseo group (black circles) after a 4-h fast in (**i**) 10-, **(j**) 14-, (**k**) 18- and (**l**) 28-week-old db/db mice (*n* = 6–8). (**m–p**) Area under the curve for glucose excursion during the OGTT in the control group (white bar) and the luseo group (black bar) in (**m**) 10-, (**n**) 14-, (**o**) 18-, and (**p**) 28-week-old db/db mice (*n* = 6–8). (**q**) Fasting plasma insulin/glucose ratio in the control group (white bar) and the luseo group (black bar) in 6-, 10-, 14-, 18- and 28-week-old db/db mice (*n* = 6–8). Values are mean ± SD. *p < 0.05; **p < 0.01.

The following experiment was performed to characterize the protective effects of luseogliflozin treatment on pancreatic beta cells in db/db mice of different ages. Male db/db mice (6, 10, 14 and 24 weeks old) were treated with or without luseogliflozin 0.01% mixed into the normal diet for 4 weeks. There were no differences in body weight between the control and luseo groups of different ages (Fig. 4a–d). Blood glucose levels of the luseo group were significantly lower than the control group regardless of age (Fig. 4e–h). Mice in the different age groups underwent an OGTT and the AUC_0–120 min_ for blood glucose levels significantly decreased in the luseo group regardless of age (Fig. 4i–p). The ratio of insulin/glucose in the control group decreased with age (Fig. 4q). Interestingly, the ratio in the luseo group was significantly higher than the control group for all ages, although the 28-week-old luseo group was significantly lower than the 10-week-old luseo group (Fig. 4q).

### Effects of luseogliflozin on beta cell morphology in db/db mice of different ages

**Figure 5:**
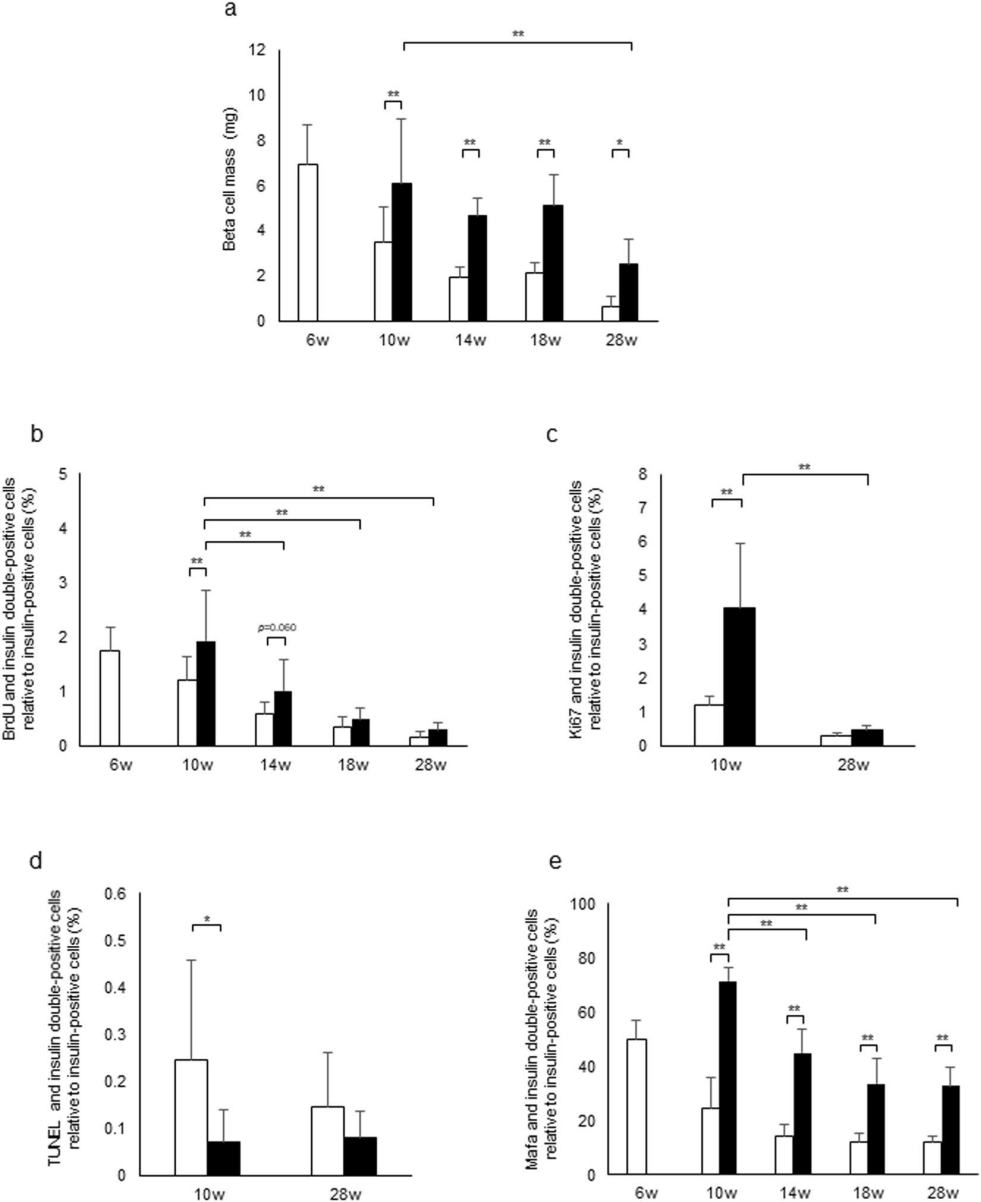
Effects of luseogliflozin on beta cell morphology in db/db mice of different ages. (**a**) Quantitation of beta cell mass in the control group (white bar) and the luseo group (black bar) in 6-, 10-, 14-, 18- and 28-week-old db/db mice (*n* = 4–8: 4–8 mice were used in each group). (**b**) Proliferation rate of beta cells assessed using BrdU incorporation in the control group (white bar) and the luseo group (black bar) in 6-, 10-, 14-, 18- and 28-week-old db/db mice (*n* = 4–8: 4–8 mice were used in each group). (**c**) Proliferation rate of beta cells evaluated using Ki67 staining in the control group (white bar) and the luseo group (black bar) in 10- and 28-week-old db/db mice (*n* = 5: five mice were used in each group). (**d**) Apoptosis rate of beta cells assessed using the TUNEL assay in the control group (white bar) and the luseo group (black bar) in 10- and 28-week-old db/db mice (*n* = 6–8: 6–8 mice were used in each group). (**e**) The ratio of the number of Mafa-positive beta cells relative to the total number of beta cells in the control group (white bar) and the luseo group (black bar) in 6-, 10-, 14-, 18- and 28-week-old db/db mice (*n* = 4: four mice were used in each group). Values are mean ± SD. * *p* < 0.05; ** *p* < 0.01.

We investigated beta cell morphology in db/db mice of different ages. Beta cell mass in the control group decreased with age (Fig. 5a). Unexpectedly, beta cell mass in the luseo group significantly increased compared with the control group regardless of age, although beta cell mass in the 28-week-old luseo group was significantly lower than the 10-week-old luseo group. Similarly, the ratio of BrdU-positive beta cells in the control group decreased with age (Fig. 5b). Although this ratio in the 10-week-old luseo group significantly increased compared with the 10-week-old control group, there were no significant differences between the luseo group and the control group for the other ages (Fig. 5b). These results were consistent with those using Ki67 staining in 10- and 28-week-old db/db mice groups (Fig. 5c).

For apoptosis, while the ratio of TUNEL-positive beta cells in the 10-week-old luseo group decreased compared with the control group, there was no difference between the two groups in 28-week-old db/db mice (Fig. 5d). Additionally, we evaluated Mafa expression in these groups (Fig. 5e) and found that the percentage of Mafa-positive beta cells in the control group decreased with age, but the percentage significantly increased in the luseo group compared with the control group regardless of age. The percentage in the 28-week-old luseo group did show a significant decrease compared with the 10-week-old luseo group (Fig. 5e). These results suggest that Mafa expression could be associated with beta cell function and beta cell mass.

## Discussion

We began by investigating the effects of luseogliflozin on beta cell function and beta cell mass in 6-week-old db/db mice, which are characteristically obese and hyperphagic, and develop diabetes at a young age. Treatment with luseogliflozin for 4 weeks improved glucose tolerance and beta cell function, and increased beta cell mass. Those observations were consistent with previous reports^13,16,17^. In db/db mice, beta cell dysfunction developed after 6 weeks’ old age (Fig. 4q). A possible reason for beta cell dysfunction could be chronic hyperglycemia followed by an increase in oxidative stress. Excessive oxidative stress resulting from chronic hyperglycemia can decrease the expression of beta cell transcription factors, such as *Mafa*, *Pdx1*^18^. We suggest that a reduction in renal glucose reabsorption with luseogliflozin could ameliorate glucose tolerance and oxidative stress, preserving beta cell function by increasing the expression of beta cell transcription factors.

Although treatment with luseogliflozin for 4 weeks increased beta cell mass in 6-week-old db/db mice (Fig. 2b), the mechanism is unclear. Studies have suggested that elevated blood glucose levels and increased beta cell proliferation were a compensatory response against insulin resistance in 4-week-old db/db mice^15^, and that nuclear exclusion of Forkhead box protein O1 (FoxO1), a key downstream mediator of glucose or insulin signaling, was a contributing factor in beta cell proliferation in 4-week-old db/db mice^15,19,20^. In 6-week-old db/db mice, beta cell proliferation was reduced along with FoxO1 nuclear translocation via hyperglycemia and increase of oxidative stress^15^. We hypothesized that luseogliflozin maintains FoxO1 in the cytoplasm, promoting beta cell proliferation in 6-week-old db/db mice. However, there was no difference in the FoxO1 nuclear expression in beta cells between the control and luseo groups in the immunofluorescence analysis (see Supplementary Fig. S1a,b). The percentage of Mafa-positive beta cells significantly increased in the luseo group compared with the control group (Fig. 3c). It is reported that mice lacking Mafa in the pancreas show a decreased beta cell mass^21^. In addition, transgenic mice that conditionally and specifically produce Mafa in db/db islet beta cells augment beta cell mass^22^. Our findings align with these results, suggesting that increased beta cell mass involves an increase in Mafa nuclear expression with luseogliflozin treatment. With regard to decreased Mafa expression due to excess of oxidative stress, Matsuoka *et al*.^23^ reported that c-Jun expression was upregulated in islets of db/db mice exposed to oxidative stress, and this expression pattern paralleled with the loss of Mafa expression. In the current study, the ratio of c-Jun-positive beta cells in the luseo group significantly decreased compared with that in the control group (see Supplementary Fig. S2a,b). This result suggests that luseogliflozin could preserve Mafa expression by suppressing oxidative stress-induced c-Jun expression, resulting in improved beta cell function and preventing decreased beta cell mass. Our finding of the association among oxidative stress, Mafa expression and beta cell mass are consistent with a study on the effects of antioxidant enzymes showing that beta cell-specific overexpression of glutathione peroxidase preserved intranuclear Mafa expression and beta cell mass in db/db mice^24^.

In addition to decreased beta cell proliferation and increased beta cell apoptosis, recent studies have suggested, as an alternative mechanism, that beta cell dedifferentiation to endocrine progenitor-like cells and conversion to other endocrine cells act to reduce beta cell mass^25–27^. We used markers of islet progenitor cells, such as neurogenin 3 (Neurog3), aldehyde dehydrogenase 1a3 (Aldh1a3), to determine whether luseogliflozin treatment could prevent beta cell dedifferentiation (see Supplementary Fig. S3a,b). There were no differences in expression levels of *Neurog3* mRNA and *Aldh1a3* mRNA, or nuclear expression levels between the control and luseo groups in 6-week-old db/db mice after 4 weeks of treatment. This was consistent with a recent study showing that the SGLT inhibitor phloridzin had no effect on the reduction of beta cell dedifferentiation^28^.

An important finding of the current study is that luseogliflozin treatment for 4 weeks improved beta cell function and increased beta cell mass in db/db mice of different ages, although improvement in 24-week-old db/db mice was to a lesser extent than that in 6-week-old db/db mice. Progressive decline of pancreatic beta cells is an established feature of T2D, and early intervention for patients with T2D is important for the protection of beta cells. Our finding of less improvement in 24-week-old db/db mice supports the “the earlier, the better” approach to treatment.

In the current study, luseogliflozin treatment improved beta cell function and increased beta cell mass in 24-week-old db/db mice, in which insulin secretion were severely impaired. A previous study on db/db mice indicated that liraglutide and/or pioglitazone treatment during the early phase of diabetes (7-week-old db/db mice) preserved beta cell function and mass, but the same treatment in the late phase (16-week-old db/db mice) did not have the same effect^29^. Similar results using liraglutide monotherapy are reported^30^. In these experiments, Mafa expression increased during intervention in the early phase of diabetes compared with the late phase^29,30^. Although the different outcomes observed between these and our own study may be related with the treatment duration or types of drugs, blood glucose decreasing potential could be an important factor contributing to the differences between the studies. In these previous reports, decreases of blood glucose following treatment in the late phase of diabetes were less than those in the early phase^29,30^. Accordingly, amelioration of glucotoxicity and Mafa expression turned insufficient, resulting in no improvement in beta cell function nor mass. To the contrary, decreases in blood glucose was almost comparable across age levels in the current study, and Mafa expression increased following luseogliflozin treatment in mice in both the early and late phases of diabetes, leading to improved beta cell function and mass even in mice in the late phase of diabetes.

This study had some limitations. First, luseogliflozin was administered for only 4 weeks. It is not shown, if more beneficial effects on beta cell function and mass could be observed in mice in the late phase of diabetes when treatment is continued from 6 to 28 weeks. Second, it remains unknown whether there are differences between the protective effects of the SGLT2 inhibitor and other antidiabetic agents on pancreatic beta cells. Future studies will compare the protective effects of insulin treatment on beta cells with that of the SGLT2 inhibitor in db/db mice, where a reduction in blood glucose with these drugs is achieved at the same level.

In conclusion, treatment with luseogliflozin preserved beta cell function and beta cell mass in db/db mice. This protective effect was observed in the early phase of diabetes and partially in the late phase. Although clinical trials to investigate the effects of luseogliflozin in patients with T2D classified by endogenous insulin secretion are needed, our findings suggest that luseogliflozin has more potential for the treatment of diabetes and related disorders than currently expected in clinical practice.

## Materials

### Animals

We used 6-, 10-, 14- and 24-week-old male BKS. Cg-Dock7^m^+/+Lepr ^db^/J (db/db) mice purchased from Oriental Yeast Co. (Tokyo, Japan). Blood glucose levels in 6-week-old db/db mice were substantially higher than those in 6-week-old littermate (db/m) mice and the percentage of Mafa-positive beta cells was significantly lower in 6-week-old db/db mice compared with 6-week-old db/m mice (data not shown). Therefore, 6-week-old db/db mice were considered as the early phase of diabetes in our study. Mice were housed two to three animals per cage for all experiments under controlled ambient conditions on a 12-h light/dark cycle (lights on at 7 a.m.). Mice were divided into two groups; fed either standard chow (CE-2, CLEA JAPAN, Tokyo, Japan) or standard chow containing luseogliflozin 0.01% for 4 weeks. Animals were given free access to drinking water and food, maintained at 25 °C. The study was approved by the Animal Use Committee of Hokkaido University Graduate School of Medicine and was conducted in compliance with the Animal Use Guidelines of the Hokkaido University.

### Compound

The SGLT2 inhibitor luseogliflozin was prepared by Taisho Pharmaceutical (Tokyo, Japan)^31^. The dosage of luseogliflozin was decided as previously described^13^.

### Measurement of biochemical parameters

Blood glucose levels were measured using a Glutestmint portable glucose meter (Sanwa Chemical Co., Nagoya, Japan). Insulin levels were measured using an insulin ELISA kit (Morinaga Institute of Biological Science, Yokohama, Japan).

### Intraperitoneal insulin tolerance test

Insulin tolerance test was performed under non-fasting conditions. Mice were injected with human regular insulin (2 units/kg body weight) intraperitoneally and measured from the tail vein at 0, 30, 60, 90 and 120 min. The total area under the blood glucose concentration curve (AUC) was calculated by trapezoidal method from time 0 to 120 min after intraperitoneal administration of insulin.

### Oral glucose tolerance test (OGTT)

For the OGTT, mice were fasted for 4 h or 16 h before being orally loaded with glucose (1.0 mg/g body weight). Blood samples were collected from the tail vein at 0, 5, 30, 60 and 120 min after glucose administration for plasma glucose levels. The total AUC was determined from 0 to 120 min.

### Immunohistochemistry

Isolated pancreatic tissues were immersion-fixed in 4% paraformaldehyde at 4 °C overnight. Tissues were then roughly paraffin-embedded and 5-μm sections were mounted on glass slides using standard procedures. Sections were immersed for 15 min in methanol containing 0.3% (vol/vol) hydrogen peroxide to deactivate endogenous peroxidase activity. After rinsing with PBS, sections were immunostained with specific antibodies, including rabbit antihuman insulin antibody (diluted 1:500) (Santa Cruz Biotechnology, Santa Cruz, CA). Sections were counterstained with hematoxylin. Beta cell mass was calculated as: beta cell mass (mg) = beta cell area/pancreatic area × pancreas weight (mg).

For immunofluorescence, tissue sections were incubated overnight at 4 °C with the primary antibodies listed in Supplementary Table 1. After rinsing with PBS, tissues were incubated with the secondary antibodies for 40 min (diluted 1:200) (see Supplementary Table 1). Immunofluorescence images were acquired using a BZ-II analyzer (Keyence, Osaka, Japan) according to the manufacturer’s instructions. At least 50 islets per mouse were counted in each group.

### Analysis of beta cell proliferation and apoptosis

Beta cell proliferation was identified by staining sections with 5-bromo-2-deoxyuridine (BrdU) antibody, as described previously^32^. Immunohistochemical detection of BrdU was performed using a commercial kit (BD Biosciences, Franklin Lakes, NJ) and was double immunostained with an anti-insulin antibody (diluted 1:500). Beta cell apoptotic cell death was identified using a fluorometric terminal deoxynucleotidyl transferase-mediated dUTP nick-end labelling (TUNEL) system (Deadend; Promega, Madison, WI). BrdU-positive and TUNEL-positive beta cells were quantitatively assessed as the percentage of the total number of beta cells and at least 50 islets per mouse were counted in each group.

### Islet isolation study

Islets were isolated using collagenase from clostridium histolyticum (Sigma-Aldrich) according to the manufacturer’s instructions. To measure insulin content, isolated islets were extracted in acid ethanol and the insulin concentration of the assay buffer was measured using an insulin ELISA kit (Morinaga Institute of Biological Science).

### Real-time quantitative PCR

Total RNA was isolated using a RNeasy mini kit (Qiagen, Hilden, Germany) and was used as the starting material for cDNA preparation. A real-time PCR study was performed in duplicate using a 7500 Fast Real Time PCR system with SYBR Green PCR Master Mix (Applied Biosystems, Foster City, CA). The results were quantified using the comparative cycle threshold method, and expression was normalized to glyceraldehyde 3-phosphate dehydrogenase (GAPDH). The primer sequences used are listed in Supplementary Table 2.

### Statistical analysis

Data are expressed as mean ± standard deviation (n). The Student’s *t*-test was used for comparisons between two groups. Individual comparisons between more than two groups were assessed using one-way analysis of variance (ANOVA) followed by the post-hoc Fisher’s partial least significant difference test. A *p*-value < 0.05 was considered statistically significant.

## Electronic supplementary material

Supplemental Table, Figure Legends, Figure
