# Supplementary material for: Effect of the sodium–glucose cotransporter 2 inhibitor luseogliflozin on pancreatic beta cell mass in db/db mice of different ages
Source: Sci Rep. 2018 May 1;8:6864. doi: 10.1038/s41598-018-25126-z (PMC5931598; doi:10.1038/s41598-018-25126-z)

Effect of the sodium–glucose cotransporter 2 inhibitor luseogliflozin on pancreatic beta cell mass in db/db mice of different ages

Kiyohiko Takahashi, Akinobu Nakamura, Hideaki Miyoshi, Hiroshi Nomoto, Naoyuki Kitao, Kazuno Omori, Kohei Yamamoto, Kyu Yong Cho, Yasuo Terauchi, Tatsuya Atsumi

Supplementary Table 1. Primary and secondary antibodies used in immunofluorescence

Primary antibodies

**Antigen**	**Species**	**Manufacturer**	**Working dilution**
Aldh1a3	Goat	Santa Cruz Biotechnology	1:200
c-Jun	Rabbit	Cell Signaling TECHNOLOGY	1:200
FoxO1	Rabbit	Cell Signaling TECHNOLOGY	1:50
Glucagon	Mouse	SIGMA-ALDRICH	1:500
Insulin	Rabbit	Santa Cruz Biotechnology	1:250
Insulin	guinea pig	Dako	1:2
Ki67	Rabbit	Cell Signaling TECHNOLOGY	1:200
Mafa	Rabbit	BETHYL	1:50

Secondary antibodies

**Antigen**	**Species**	**Fluorescent dye**	**Manufacturer**
guinea pig IgG	Goat	Alexa Fluor 488	Life Technologies
rabbit IgG	Goat	Alexa Fluor 488	Life Technologies

rabbit IgG	Donkey	Alexa Fluor 488	abcam
rabbit IgG	Goat	Alexa Fluor 594	Life Technologies
mouse IgG	Goat	Alexa Fluor 594	Life Technologies
goat IgG	Donkey	Alexa Fluor 594	Abcam

Supplementary Table 2. The primer sequences used for real-time quantitative PCR

Gene (Forward/Reverse)	Sequence
GAPDH forward	GGCCCCTCTGGAAAGCTGTGGTGT
GAPDH reverse	GTTGGGGGCCGAGTTGGGATAGG
Mafa forward	CTTCAGCAAGGAGGAGGTCATC
Mafa reverse	GCGTAGCCGCGGTTCTT
Pdx1 forward	CTCCGGACATCTCCCCATAC
Pdx1 reverse	ACGGGTCCTCTTGTTTTCCT
NKX6.1 forward	CTGCACAGTATGGCCGAGATG
NKX6.1 reverse	CCGGGTTATGTGAGCCCAA
Ins1 forward	GACCAGCTATAATCAGAGACC
Ins1 reverse	AGTTGCAGTAGTTCTCCAGCTG
Ins2 forward	AGCCCTAAGTGATCCGCTACAA
Ins2 reverse	AGTTGCAGTAGTTCTCCAGCTG
Gck forward	AGAAGGCTCAGAAGTTGGAGAC
Gck reverse	GGATGGAATACATCTGGTGTTTCG
Glut2 forward	TGTGGTGTCGCTGTTTGTTG
Glut2 reverse	AATGAAGTTTGAGGTCCAGTTGG
Ki67 forward	CTGCCTGCGAAGAGAGCATC
Ki67 reverse	AGCTCCACTTCGCCTTTTGG
Ccnd1 forward	TAGGCCCTCAGCCTCACTC
Ccnd1 reverse	CCACCCCTGGGATAAAGCAC
Ccnd2 forward	AAGCCTGCCAGGAGCAAA
Ccnd2 reverse	ATCCGGCGTTATGCTGCTCT
Ccnd3 forward	CCAGCGTGTCCTGCAGAGTT
Ccnd3 reverse	CCTTTTGCACGCACTGGAAG
p22phox forward	TGCCAGTGTGATCTATCTGCT
p22phox reverse	TCGGCTTCTTTCGGACCTCT
gp91phox forward	AGCTATGAGGTGGTGATGTTAGTGG
gp91phox reverse	CACAATATTTGTACCAGACAGACTTGAG
p47phox forward	ACACCTTCATTCGCCATATTGC
p47phox reverse	CCTGCCACTTAACCAGGAACA
Neurog3 forward	CCAAGAGCGAGTTGGCACT
Neurog3 reverse	CGGGCCATAGAAGCTGTGG
Aldh1a3 forward	ATCAACAACGACTGGCACGAA
Aldh1a3 reverse	CACATCGGGCTTATCTCCTTC

Supplementary Figure Legends

Supplementary Figure S1. Localization of FoxO1 expression in beta cells of 6-week-old db/db mice with or without luseogliflozin for 4 weeks

a: Representative insulin (green) and FoxO1 (red) staining in pancreas sections from the control group and the luseo group. b: Percentage of FoxO1 nuclear expression in beta cells in the control group (white bar) and the luseo group (black bar) (*n*=4: four mice were used in each group). Values are mean ± SD. Scale bars: 50 μm.

Supplementary Figure S2. Changes in c-Jun-positive beta cells in islets of 6-week-old db/db mice with or without luseogliflozin for 4 weeks

a: Representative insulin (green) and c-Jun (red) staining in pancreas sections from the control group and the luseo group. b: Ratio of the number of c-Jun-positive beta cells relative to the total number of beta cells in the control group (white bar) and the luseo group (black bar) (*n*=4: four mice were used in each group). Values are mean ± SD. ** $p<0.01$. Scale bars: 50 μm.

Supplementary Figure S3. Changes in beta cell dedifferentiation using markers of islet progenitor cells in 6-week-old db/db mice with or without luseogliflozin for 4 weeks

a: Gene expression levels of *Neurog3* and *Aldh1a3* in islets measured using real-time quantitative PCR. Data have been normalized to GAPDH expression (*n*=3-4). b: Representative insulin (green) and Aldh1a3 (red) staining in pancreas sections from the control group and the luseo group. Values are mean ± SD. Scale bars: 50 μm.

Supplementary Figure S1

a

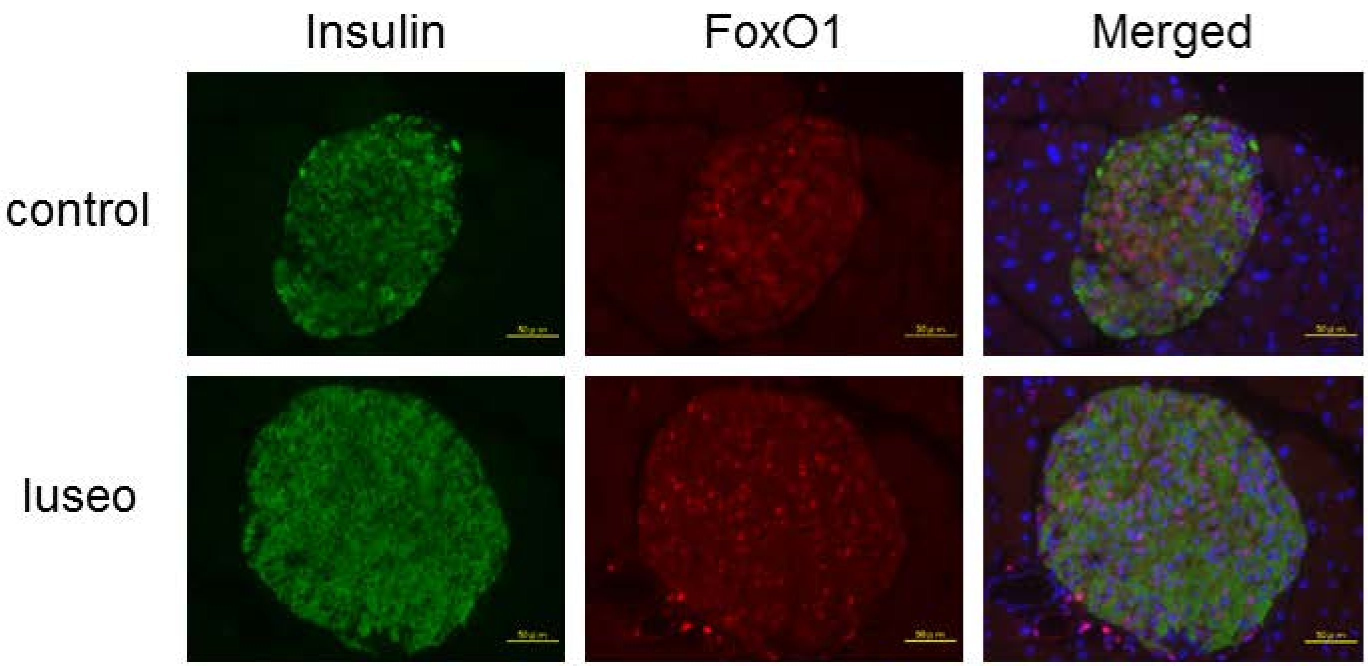

b

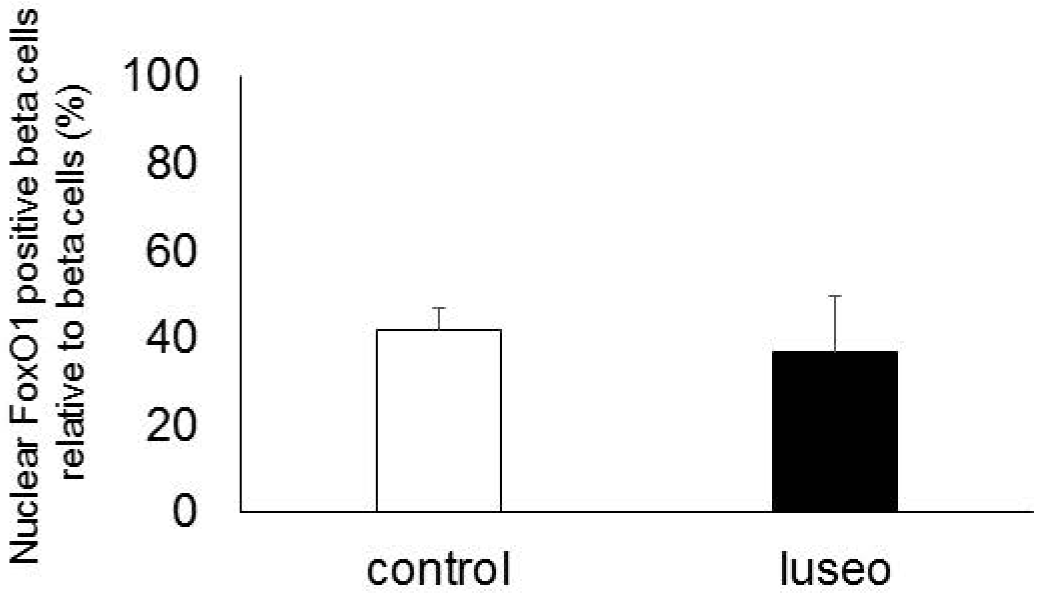

Supplementary Figure S2

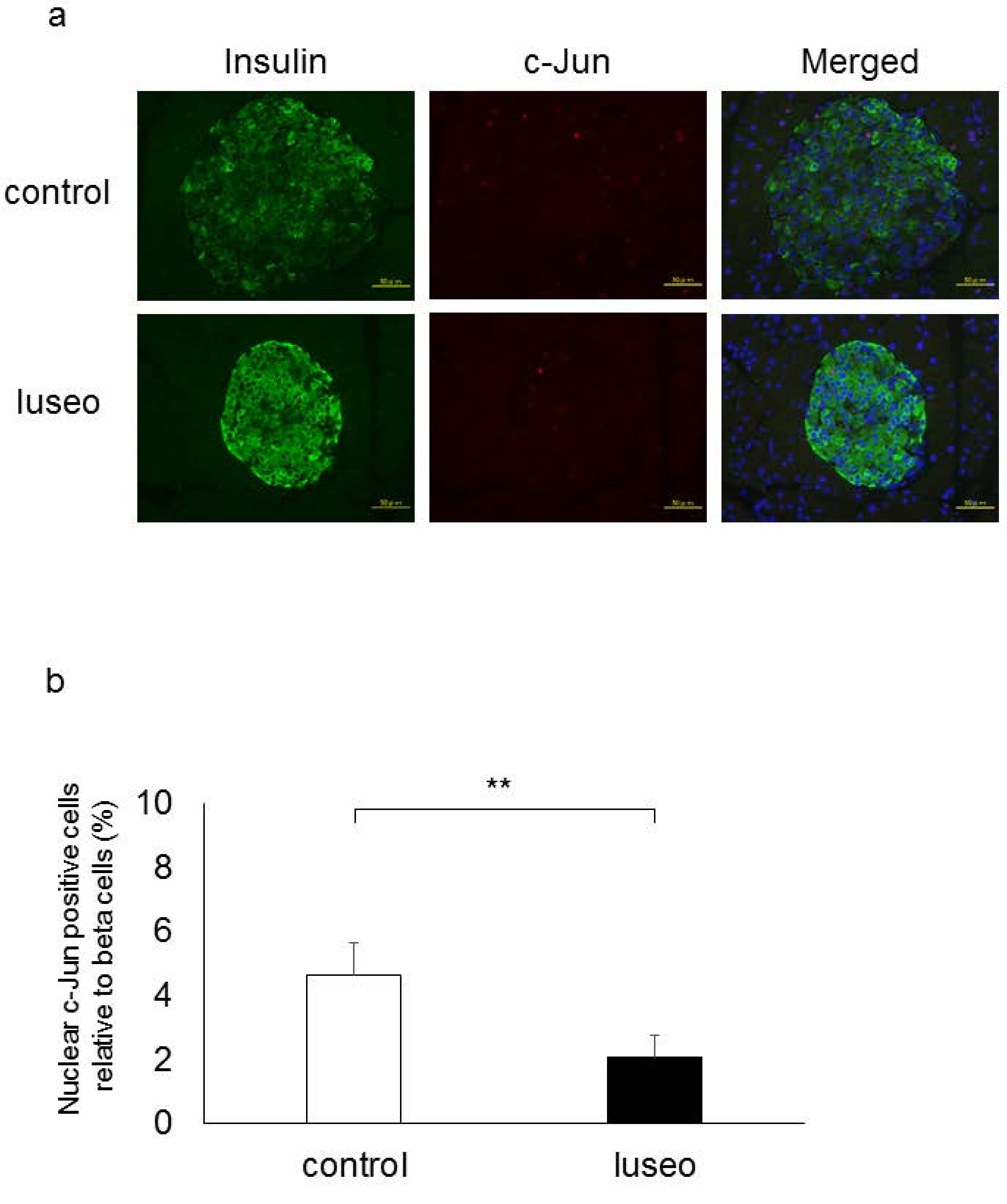

Supplementary Figure S3

a

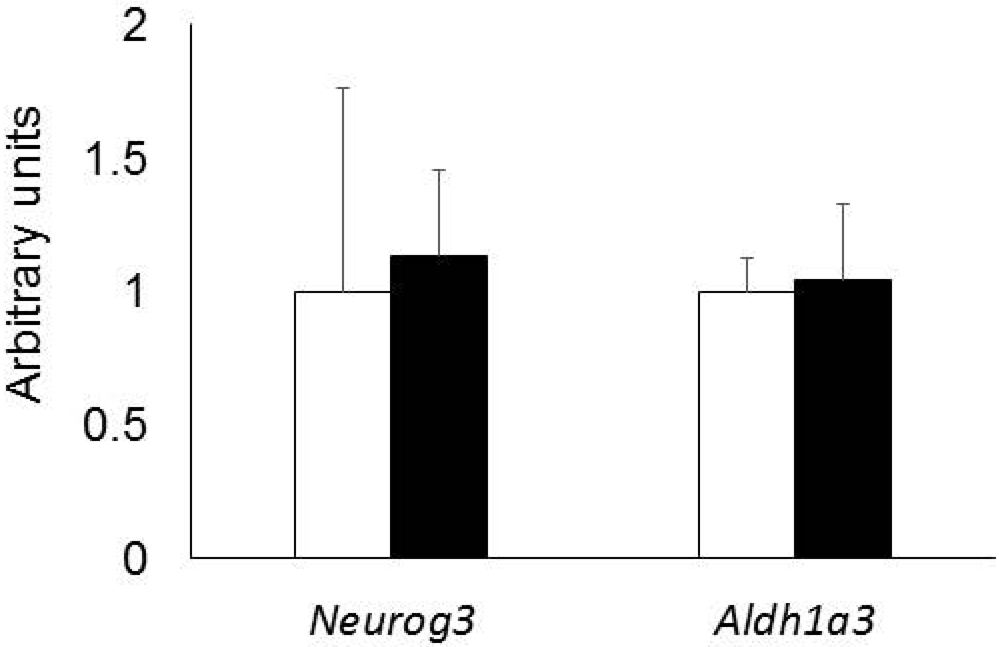

b

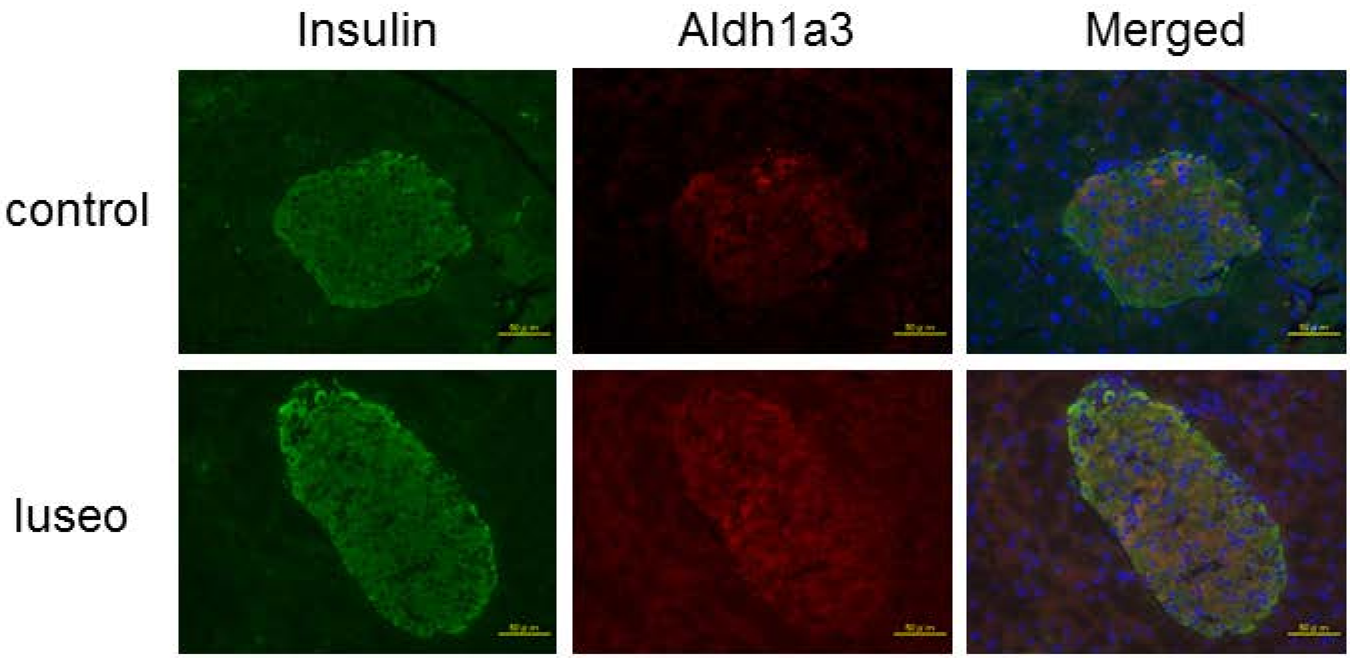

Supplement: Supplementary file 1 — Supplemental Table, Figure Legends, Figure [file 41598_2018_25126_MOESM1_ESM.pdf]
